# MRGPRX4 mediates phospho-drug associated pruritus in a humanized mouse model

**DOI:** 10.1126/scitranslmed.adk8198

**Published:** 2024-05-08

**Authors:** Daphne Chun-Che Chien, Nathachit Limjunyawong, Can Cao, James Meixiong, Qi Peng, Cheng-Ying Ho, Jonathan F. Fay, Bryan L. Roth, Xinzhong Dong

**Affiliations:** 1 Solomon H. Snyder Department of Neuroscience, Johns Hopkins University School of Medicine, Baltimore, MD 21205, USA.; 2 Department of Pharmacology, University of North Carolina at Chapel Hill School of Medicine, Chapel Hill, NC 27599, USA.; 3 Department of Dermatology, University of California San Francisco, San Francisco, CA 94115, USA.; 4 Department of Pathology, Johns Hopkins University School of Medicine, Baltimore, MD 21205, USA.; 5 Department of Biochemistry and Molecular Biology, University of Maryland Baltimore, Baltimore, MD 21201, USA; 6 Department of Dermatology, Johns Hopkins School of Medicine, Baltimore, MD 21205, USA.; 7 Department of Neurosurgery, Johns Hopkins University School of Medicine, Baltimore, MD 21205, USA.; 8 Howard Hughes Medical Institute, Chevy Chase, MD 20815, USA.

## Abstract

The phosphate modification of drugs is a common chemical strategy to increase solubility and allow for parenteral administration. Unfortunately, phosphate modifications often elicit treatment- or dose-limiting pruritus through an unknown mechanism. Using unbiased high-throughput drug screens, we identified the Mas-related G protein-coupled receptor X4 (MRGPRX4), a primate-specific, sensory neuron receptor previously implicated in itch, as a potential target for phosphate-modified compounds. Using both Gq-mediated calcium mobilization and G protein-independent GPCR assays, we found that phosphate-modified compounds potently activate MRGPRX4. Furthermore, a humanized mouse model expressing MRGPRX4 in sensory neurons exhibited robust phosphomonoester prodrug-evoked itch. To characterize and confirm this interaction, we further determined the structure of MRGPRX4 in complex with a phosphate-modified drug through single-particle cryogenic electron microscopy (cryo-EM) and identified critical amino acid residues responsible for the binding of the phosphate group. Together, these findings explain how phosphorylated drugs can elicit treatment-limiting itch and identify MRGPRX4 as a potential therapeutic target to suppress itch and to guide future drug design.

## One Sentence Summary

Phosphate modification of medications triggers pruritus through the sensory neuron receptor MRGPRX4.

## INTRODUCTION

Phosphate addition is a common medicinal chemistry strategy to increase drug solubility and has been employed to both facilitate parenteral routes of numerous agents and to permit the administration of a larger quantity of drugs using a smaller injection volume (1). For example, the addition of phosphate to dexamethasone to create dexamethasone sodium phosphate, enhances its aqueous solubility by more than 500-fold (2). This modification allows for pre-operative intravenous (IV) bolus administration, which is associated with decreased postoperative complications. IV dexamethasone sodium phosphate and other phospho-ester prodrugs are preferable in select clinical scenarios; however, the usage of these medications has been associated with distressing pruritus and dysesthesias as well as other cutaneous adverse drug reactions (3–8) for which the mechanism remains elusive.

Over the past two decades, the human MRGPRX subfamily (X1-X4) of class A G protein-coupled receptors (GPCRs) has garnered considerable attention as itch-associated receptors (9–17). Our lab and others have identified numerous ligands, both endogenous and exogenous, which activate these receptors and are known to be pruritogenic (11–17). Despite the lack of conserved sequences in species other than human or non-human primates (18–20), several pairs of mouse-human receptors have been identified through shared ligand activation (21). Of note, murine receptors found to share ligand activation with human MRGPRX subfamily are concentrated within the *MrgprA-C* clusters, which contain approximately 30 genes with intact coding sequences (9, 22). For example, chloroquine, an anti-malaria drug with prominent itch side effect, was found to activate both human MRGPRX1 and mouse Mrgpra3 (11).

MRGPRX4 is a Gq-coupled GPCR expressed specifically in small-diameter peripheral itch-sensing neurons of the dorsal root ganglia (DRG) and trigeminal ganglia (TG) (12, 15–17, 23, 24). These neurons are first order neurons of the somatosensory afferent pathway which convey neuronal signal to the central nervous system to generate itch perception (25–29). Previously, our group and others have identified MRGPRX4 as being a bile acid and bilirubin receptor which potentially mediates a component of cholestatic itch as well as a receptor for nateglinide, an anti-diabetic drug that causes itch (15–17, 30). Given these previously identified ligands, their clinical implication, and the burden of iatrogenic itch, we initiated a non-biased high-throughput drug screen against MRGPRX4 and identified a class of agents, all sharing a key structural phosphate component, which activate the receptor. In a mouse model expressing MRGPRX4, phosphate-containing drugs elicited MRGPRX4-dependent itch. To better characterize this interaction and guide drug development, we determined the structure of a phosphomonoester drug-bound MRGPRX4, which provided structural insight into drug-receptor interactions. Together, these findings suggest that MRGPRX4 mediates phospho-drug evoked itch and provide a potential therapeutic target to reverse this unintended side effect.

## RESULTS

### High throughput screening of medications identifies a class of phosphomonoester prodrugs as MRGPRX4 ligands

**Fig. 1.**
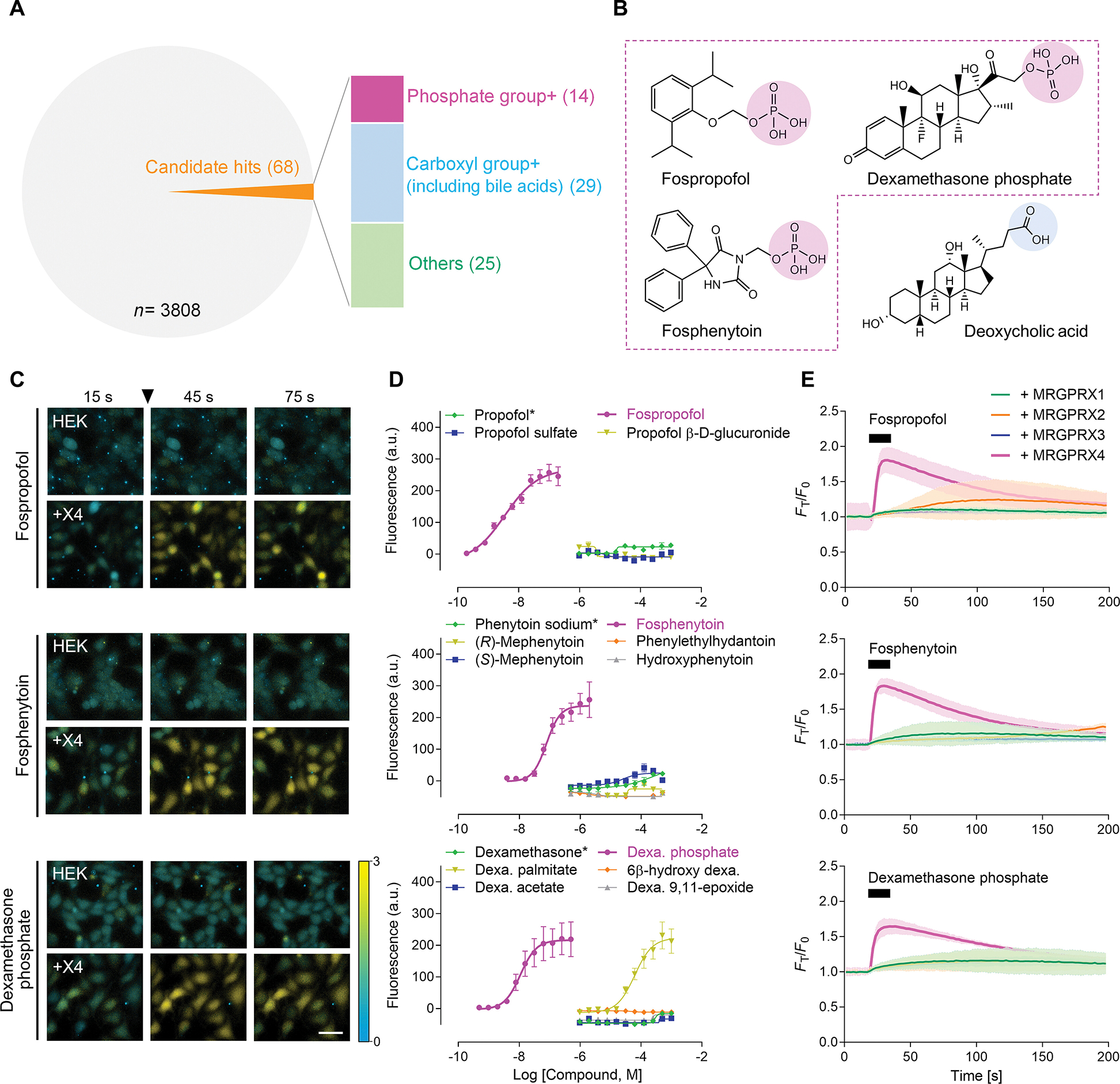
Intracellular calcium mobilization assays identify phosphate monoester prodrugs activating MRGPRX4. (**A**) Phosphate drugs were among the candidate drugs that elicited calcium response in MRGPRX4-expressing HEK293 cells. Hits were determined by a cutoff of Z-score ≥ 3. (**B**) Structure of key phosphomonoester drugs (outlined in dashed magenta) and deoxycholic acid (bile acids), a previously established MRGPRX4 agonist. Phosphate and carboxyl group are circled in magenta and blue, respectively. (**C**) Representative images of intracellular [Ca^2+^] change of HEK293 cells (HEK) and MRGPRX4-expressing HEK293 cells (+X4) responding to 10 μM fospropofol (*top*), fosphenytoin (*middle*), and dexamethasone phosphate (*bottom*). Compounds were added at 30 s after recording started as pointed by arrowheads. Scale bar, 20 μm. Intensity of intracellular [Ca^2+^] change was measured with changes in Fura-2 fluorescence 340/380 ratio. (**D**) Dose-response curves of fospropofol (*top*), fosphenytoin (*middle*), and dexamethasone phosphate (*bottom*) with their parental drug (*) and other conjugations. Data are from three independent experiments, each performed in triplicate, and are reported as the mean ± SEM. (**E**) Time course of Fura-2 fluorescence 340/380 ratio responding to 500 μM fospropofol (*top*), fosphenytoin (*middle*), and dexamethasone phosphate (*bottom*) from HEK293 cells overexpressing MRGPRX1-X4. Black bars indicate the application of each drug. Data are from two independent experiments, each performed in duplicate, and are reported as mean ± 95% confidence interval (shaded areas).

MRGPRX4 engages intracellular Gq to induce calcium flux (15, 17, 23). Using calcium imaging as a readout, we screened 3,808 drugs for activity against human embryonic kidney (HEK) 293 cells expressing MRGPRX4 (the Serine 83, rs2445179 variant). Because *MRGPRX4* is highly polymorphic (20, 31–33), the NCBI gene reference sequence does not annotate the dominant allele. Given this, we utilized the NCBI single nucleotide polymorphism database to identify the highest represented sequence, MRGPRX4 S83, which was used throughout this study unless otherwise specified. Drugs were delivered at 20 μM concentration and top candidate hits were defined as exhibiting calcium responses with Z-score ≥ 3. With these criteria, sixty-eight candidates were identified on the initial screen as displaying high affinity for MRGPRX4 (Fig. 1A). Among the identified ligands, numerous compounds contained either phosphate monoesters or carboxyl groups (including bile acids, previously identified as MRGPRX4 agonists (15, 17)) (Fig. 1A and 1B). We externally validated our initial inferences by testing phosphomonoester drugs not present in our screening library and identified fospropofol, a discontinued sedative agent, as a potent agonist of MRGPRX4. Given the published association between MRGPRX4 and carboxyl group-containing ligands such as bile acids (15–17, 23, 30), we focused our additional studies on the link between this receptor and phosphate monoester prodrugs.

To assess the physiologic relevance of these agonist-receptor interactions, we determined the EC_50_ (half of the maximal effective concentrations) values for each drug. Several phosphomonoester compounds including fospropofol (EC_50_: 3.78 nM [95% confidence interval (CI): 1.82 – 6.78]), fosphenytoin (an antiepileptic drug, EC_50_: 77.01 nM [95% CI: 52.63 – 115.10]), and dexamethasone phosphate (steroid-derived phosphate, EC_50_: 14.68 nM [95% CI: 5.44 – 22.10]) showed high agonist potencies for MRGPRX4 (Fig. 1C and D, table S1.). Given the shared phosphate moiety among many otherwise divergent chemical structures, we hypothesized that this group was crucial for ligand activation of MRGPRX4. Indeed, removal of the phosphate group abolished MRGPRX4-depedendent calcium signaling (Fig. 1D). Similarly, substitution of phosphate with other functional groups either drastically reduced or completely annulled signaling (Fig. 1D). To validate the engagement of canonical GPCR pathways through β-arrestins as a downstream effector, we verified activation of candidate drugs using PRESTO-Tango (30) and obtained similar results (fig. S1).

As an additional control, we assessed the specificity of calcium signaling through pharmacologic inhibitors of Gq (YM-254890), phospholipase C (U73122), and sarco/endoplasmic reticulum Ca^2+^-ATPases (thapsigargin). For genuine MRGPRX4-ligand interactions, we would expect these inhibitors to abrogate Ca^2+^ flux, and indeed, in all tested cases, inhibition of Gq, phospholipase C, or sarco/endoplasmic reticulum Ca^2+^-ATPases abolished the calcium response (fig. S2). The calcium response observed with phospho-ligands and MRGPRX4 was specific to the receptor. We showed that HEK293 cells expressing MRGPRX1, MRGPRX2, or MRGPRX3 were not activated by these compounds, which confirmed these drugs do not cross-react with other human MRGPRXs (Fig. 1E). Additionally, we conducted similar screens for activity of phosphate-modified drugs against the murine-expressed Mrgprs and were not able to identify any activated murine receptors including Mrgpra1, previously linked to MRGPRX4 due to shared activation by bilirubin (16) (fig. S3). Overall, these results suggest that a common chemical feature among phosphorylated drugs leads to their interaction with a single receptor, MRGPRX4.

### Humanized MRGPRX4-expressing mice exhibit phosphomonoester drugs-associated pruritus

Clinically, fosphenytoin, fospropofol, and steroid phosphates have all been associated with pruritus as 48.9% of patients who received IV fosphenytoin in clinical trials reported itch compared to 4.5% of patients who received IV phenytoin (New Drug Application: 020450). Additionally, 26.0% and 53.6% of patients who received fospropofol reported pruritus and paresthesia, respectively (ClinicalTrials.gov Identifier: NCT00327392). Given previous association with pathophysiologic, non-histaminergic itch, we reasoned that MRGPRX4 may be the convergent receptor mediating pruritus triggered by these drugs.

**Fig. 2.**
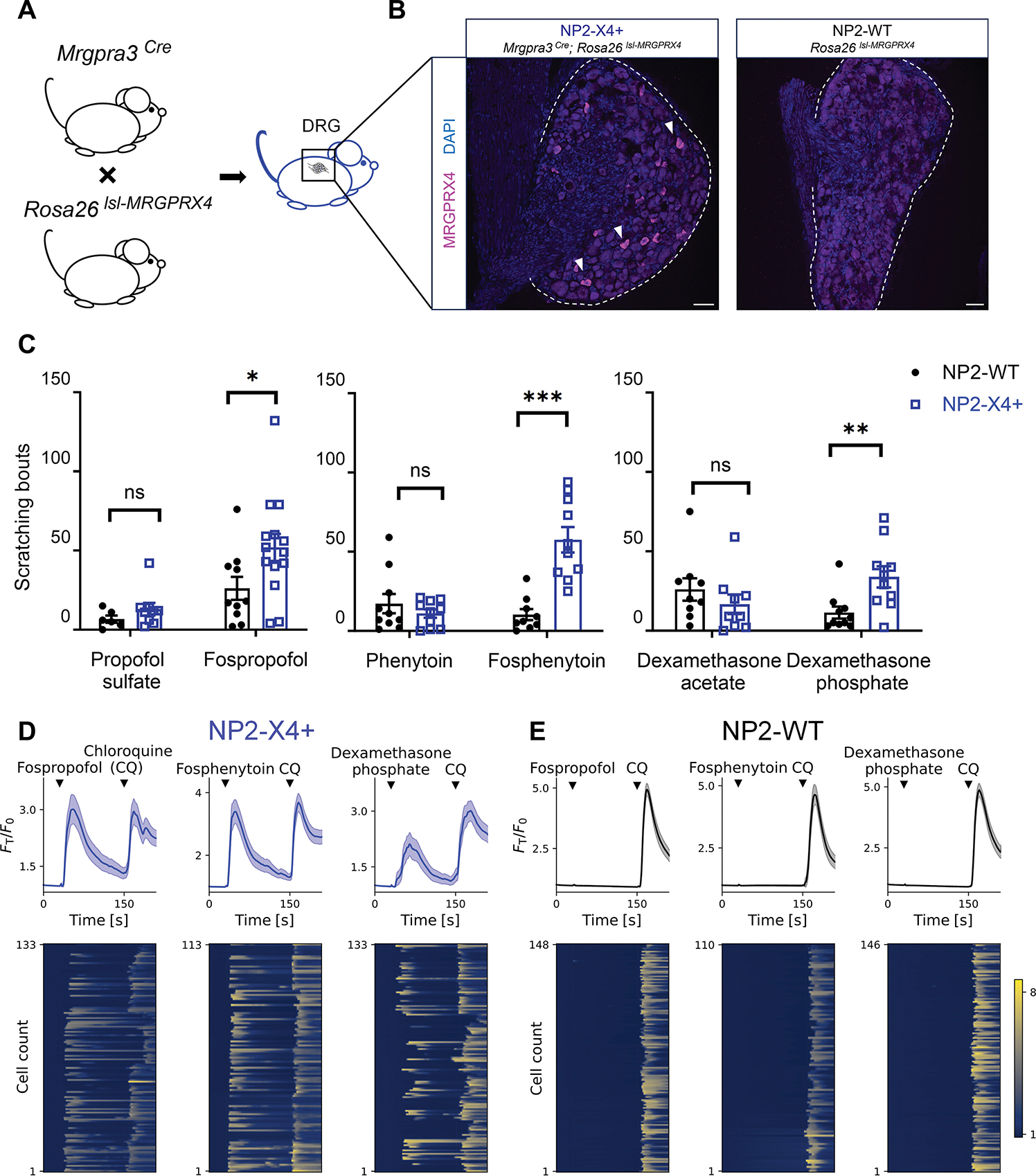
Phosphate monoester prodrugs induce itch in NP2-MRGPRX4+ mice through activating NP2 primary sensory neurons. (**A**) Mating strategy to generate NP2-X4+ mice. **(B)** In situ hybridization showing *MRGPRX4* transcripts (magenta) in NP2-X4+ DRG as indicated by arrowheads (*left*) but not in littermate control NP2-WT (*right*). NP2 subpopulation accounts for 5% of total DRG neurons. DAPI, blue. Scale bar, 50 μm. (**C**) Number of scratching bouts in 30 min after injection of 50 μl 10 mM propofol sulfate *vs*. fospropofol (*left*), phenytoin *vs*. fosphenytoin (*middle*), or dexamethasone acetate *vs*. dexamethasone phosphate (*right*) into the nape of NP2-X4+ or littermate control (NP2-WT) animals (n = 6–15 mice per group). Data are expressed as mean ± SEM. *p< 0.05, **p< 0.01 and ***p< 0.001 by two-tailed unpaired Student’s t-test. ns = no statistical significance observed. (**D and E**) Traces of average (*top*, depicted as mean ± 95% confidence interval) and individual (*bottom*, reflected by the heatmap) calcium activity, measured by fluorescence intensity normalized to the baseline intensity (F_T_/F_0_) over time of NP2-X4+ (**D**) and NP2-WT (**E**) DRG neurons labelled with GCaMP6s (n = 110–150 neurons pooled from 3–7 independent culture vessels from 2–4 mice in each condition). 10 μM phospho-drugs and 1 mM CQ were added as indicated by the arrowheads. Neurons with F_T_/F_0_ > 1.5 after compound application were considered a positive responder. NP2-X4+: NP2-MRGPRX4+; DRG, dorsal root ganglia; CQ: chloroquine.

In human dorsal root ganglia (DRG), MRGPRX4 is expressed by the “non-peptidergic neuron 2 (NP2)” sensory neuron population, which shares a similar gene expression profile to the mouse NP2 neuron population, a subtype of non-peptidergic itch-sensing neurons (24). Because no murine ortholog was identified in our screen, mechanistic assessment of behavior required a humanized mouse expressing MRGPRX4 in the corresponding murine sensory neuron population. In mice, itch-sensing NP2 neurons constitute approximately 5% of total sensory neurons in DRG and can be identified by expression of the marker gene *Mrgpra3* (10, 11, 34–36). To assess whether phospho-drug-MRGPRX4 interactions trigger itch, we crossed *Mrgpra3-Cre* mice to *Rosa26-lsl-MRGPRX4* mice as previously developed (15) (Fig. 2A) to express MRGPRX4 under the control of the *Mrgpra3* promoter and thus within murine NP2 neurons (referred to as NP2-X4+). (Fig. 2B).

When injected with fospropofol, fosphenytoin, and dexamethasone phosphate, NP2-X4+ mice, but not their WT littermates, displayed significantly increased scratching behavior (p <0.05 for fospropofol, p < 0.001 for fosphenytoin, and p <0.01 for dexamethasone phosphate) (Fig. 2C and fig. S4). As a negative control, we assessed whether non-phosphorylated, soluble cognates (phenytoin, propofol sulfate, dexamethasone acetate) could induce itch. When injected into NP2-X4+ and littermate WT mice, no differences in itch behavior were detected (Fig. 2C). Of note, dexamethasone acetate was associated with slightly elevated itch in both NP2-X4+ and littermate WT controls, a result which we hypothesize was due to solubility issue causing local skin irritation.

Given that the initial drug screen was conducted with heterologous MRGPRX4-overexpressing HEK 293 cells, which can display aberrant signaling, we sought to confirm calcium dynamics within sensory neuron populations. We labeled NP2 neurons from NP2-WT and NP2-X4+ animals with GCaMP6, a genetically encoded calcium indicator (fig. S5) and assayed for activity associated with phosphate-containing drugs.

Fospropofol, fosphenytoin, and dexamethasone phosphate triggered strong calcium transients in NP2-X4+ neurons (Fig. 2D) but not NP2-WT neurons (Fig. 2E). Chloroquine (CQ, agonist for Mrgpra3 (11), known to activate NP2 neurons) was added subsequently as positive control and indeed, NP2-X4+ neurons and NP2-WT neurons responded to application of this drug (Fig. 2D and E). NP1 and NP3 populations have also been linked to itch by expressing receptors of various pruritogens (34–36). To exclude a role for NP1–3 populations in phosphate-modified drug-evoked itch, we performed dissociated murine DRG culture from *Pirt-Cre; Rosa-lsl-GCaMP6* animals which express GCaMP6 in nearly all primary sensory neurons (37). The populations NP1–3 were identified based on their calcium responses to different pruritogens, coupled by their cognate receptors. β-alanine activates the NP1 population through Mrgprd, and histamine activates NP2 and NP3 through the H1 histamine receptor (fig. S6A) (35, 38). Among the NP1–3 populations, few neurons were activated by either fospropofol, fosphenytoin, or dexamethasone phosphate (fig. S6B and C). These results demonstrated that the phosphomonoester prodrug-evoked itch is specifically mediated by the activation of MRGPRX4 in humanized MRGPRX4-expressing mice.

### Cryo-EM structure of ligand-bound MRGPRX4 Gq complex reveals a phosphate binding pocket

No previously identified MRGPRX4 agonist contains a phosphate group. Given the structural differences between the phosphate-modified drugs and the previously identified MRGPRX4 agonists, we next investigated the binding mode of phosphate drugs at MRGPRX4 using cryo-EM. As fospropofol is the most potent drug obtained from the screen, we used fospropofol to assemble the agonist-bound MRGPRX4-Gq complex and determined the structure at a nominal resolution of 3.1 Å (fig. S7).

**Fig. 3.**
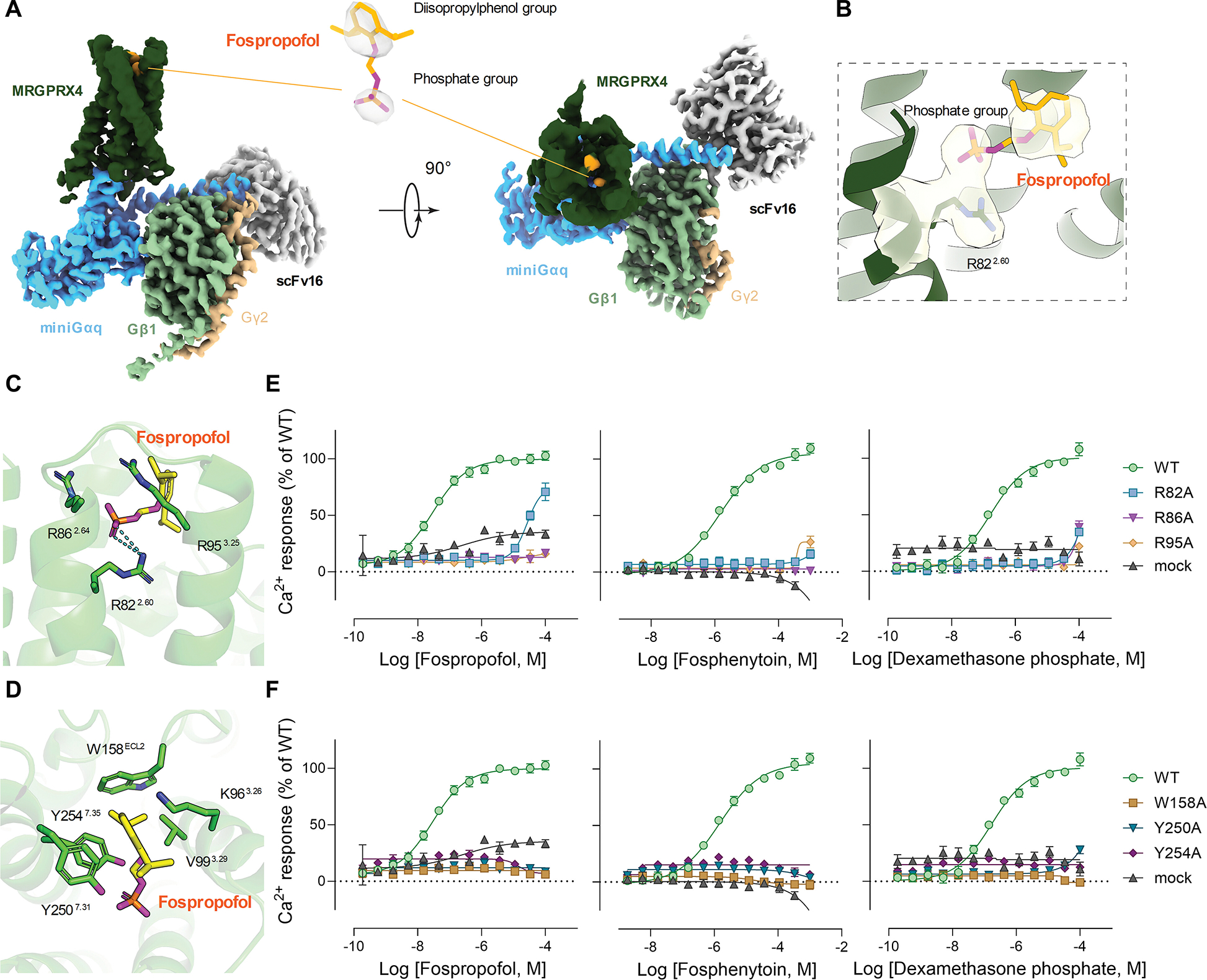
Cryo-EM structure of fospropofol-bound MRGPRX4-Gq complex identifies key amino acid residues. **(A)** Cryo-EM density map of MRGPRX4-Gq in complex with fospropofol. **(B)** The density map of fospropofol and R82 of MRGPRX4. **(C** and **D)** Key molecular interactions between fospropofol and the hydrophilic **(C)** and hydrophobic **(D)** part of the agonist binding pocket of MRGPRX4. Amino acid residues interacting with fospropofol are shown as sticks. Hydrogen bond is shown as cyan dashed lines. **(E** and **F)** Dose-response curves from calcium mobilization assay of fospropofol (*left*), fosphenytoin (*middle*), or dexamethasone phosphate (*right*) toward alanine mutant of residues forming hydrophilic (phosphate) **(E)** or hydrophobic **(F)** binding pocket. Data are from at least three independent experiments, each performed in triplicate, and reported as the mean ± SEM. **(E)** and **(F)** were collected from the same sets of experiments. Hence, WT and mock are shared between both panels.

Due to the overall shallow binding mode of MRGPR members to their agonists (23, 33, 39), it is usually challenging to obtain a cryo-EM map of MRGPR protein with a clear ligand density. Although only part of the fospropofol density was observed in the MRGPRX4 structure, an apparent density corresponding to the phosphate group was resolved (Fig. 3A). We then modeled fospropofol into the cryo-EM map according to ligand’s map density and geometry. In the refined model, the phosphate group of fospropofol formed strong charge interactions with R82 of MRGPRX4 (Fig. 3B). In proximity to R82, the orthosteric pocket of MRGPRX4 contains two additional arginine residues, R86 and R95 (Fig. 3C). Although their side chains were not fully resolved in our structure, these two residues may also bind phosphate group based on our previous observations (23). At the other end of the ligand, the diisopropylphenol group of fospropofol is inserted into a hydrophobic pocket mainly formed by residues V99, W158, Y250, and Y254 of MRGPRX4 (Fig. 3D).

Structurally, all the newly identified MRGPRX4 agonist compounds consist of both a phosphate group and a large hydrophobic group. To test if the observed polar and hydrophobic interactions are important for fospropofol-stimulated MRGPRX4 activation, we performed mutagenesis studies of MRGPRX4 using fluorometric imaging plate reader (FLIPR) calcium assay. Our result showed that alanine substitution of R82, R86, and R95 either greatly attenuated or abolished calcium mobilization responses to fospropofol, fosphenytoin, and dexamethasone phosphate. This data provided evidence that these positively charged residues are necessary for MRGPRX4 to recognize the negatively charged phosphate group (Fig. 3E and table S3). Correspondingly, alanine substitution of residues forming the hydrophobic pocket (W158, Y250, and Y254) also disrupted calcium mobilization, not only by fospropofol but also by fosphenytoin and dexamethasone phosphate (Fig. 3F and table S3), suggesting hydrophobic interactions are also critical in the recognition of the hydrophobic substructure within phosphorylated drugs.

### The MRGPRX4 predominant variant S83 demonstrates higher sensitivity toward phosphate-modified drugs

MRGPRX4 is a highly polymorphic protein with many missense mutants reported. Of note, the previously published cryo-EM structure of MS47134-bound MRGPRX4 Gq complex (23) used the S83L variant because it is the reference coding sequence annotated in NCBI database. Here, we used S83-MRGPRX4 for structural determination as this variant accounts for 99% of the human population.

**Fig. 4.**
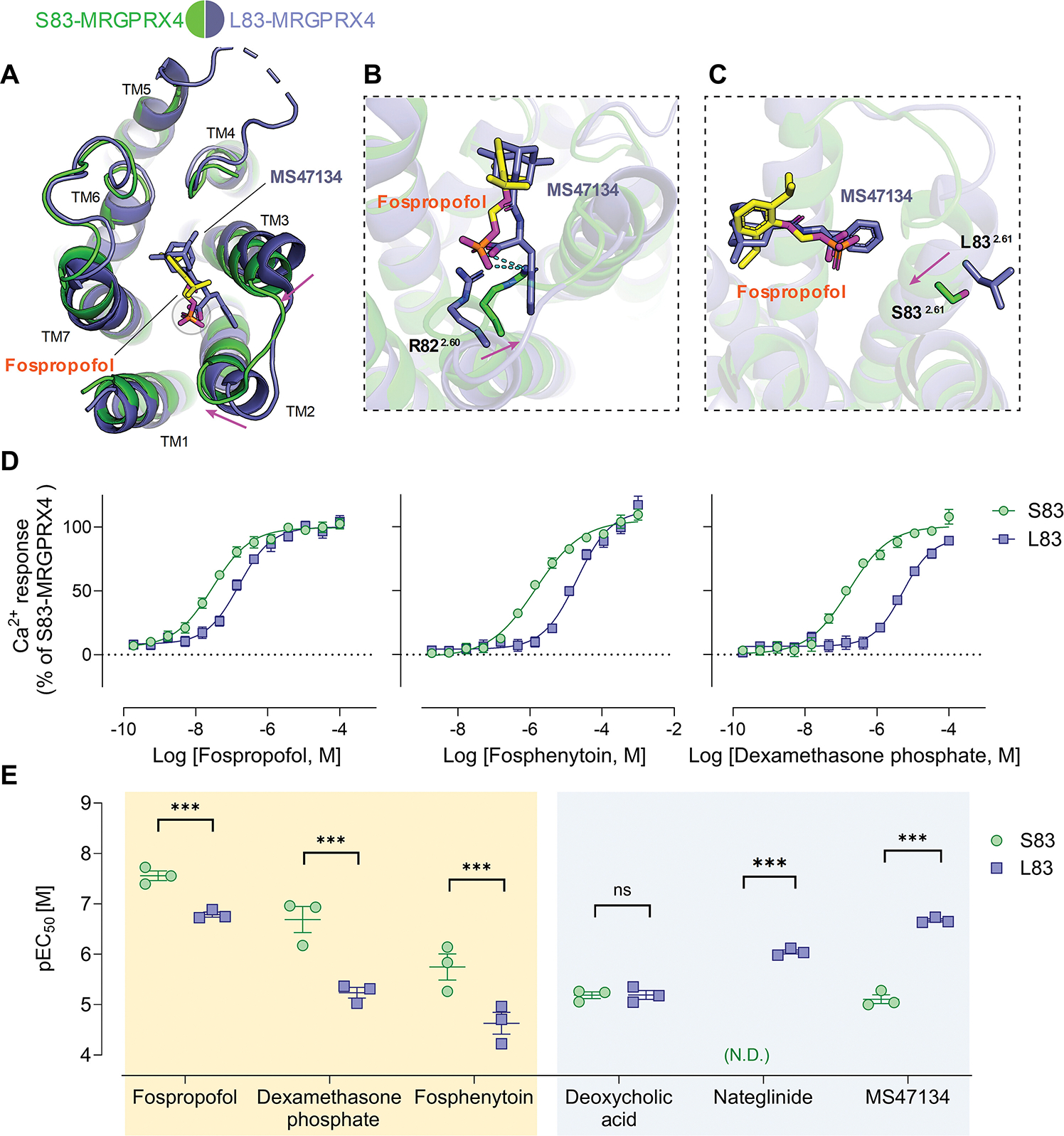
Structural and pharmacological differences of S83- and L83-MRGPRX4 variants. **(A)** Structural comparison of fospropofol-bound S83-MRGPRX4 and MS47134-bound L83-MRGPRX4 (PDB: 7S8P). Phosphate group overlays with the carboxyl group as circled. The relative movements of helices are indicated by magenta arrows. **(B-C)** Zoomed-in view of the agonist binding pocket to show the relative conformational changes of residue R82 **(B)** and residue S/L83 upon binding to fospropofol **(C)**. Hydrogen bond is shown as cyan dashed lines. **(D)** Dose-response curves from calcium mobilization assay of fospropofol (*left*), fosphenytoin (*middle*), or dexamethasone phosphate (*right*) toward S83-*vs.* L83-MRGPRX4 transiently expressed in HEK293 cells. Data are from three independent experiments, performed in triplicate. **(E)** pEC_50_ plots of various agonists toward S83- and L83-MRGPRX4 through calcium mobilization assay. Phospho-drugs (fospropofol, dexamethasone phosphate, and fosphenytoin) and carboxyl-group-containing compounds (deoxycholic acid, nateglinide, and MS47134) are boxed in yellow and blue separately. Each data point represents an independent experiment, performed in triplicate. The extra sum-of-square F test was used to examine the difference in EC_50_ values. One-sided t-test was used for nateglinide. *p< 0.05, **p< 0.01 and ***p< 0.001. ns = no statistical significance. Both **(D)** and **(E)** are reported as the mean ± SEM. N.D.= no detectable response ≤ 100 μM.

Compared with MS47134-bound L83-MRGPRX4 structure (PDB: 7S8P) (23), fospropofol binds to MRGPRX4 at a position similar to MS47134. In addition, the phosphate group of fospropofol overlaid well with the carboxyl group of MS47134 (Fig. 4A to 4C), suggesting MRGPRX4 has a conserved binding site for the negatively charged groups. However, the binding of fospropofol to S83-MRGPRX4 also causes large conformational changes in the agonist binding pocket. Both transmembrane helix 2 and 3 (TM2/3) of fospropofol-bound MRGPRX4 display an inward movement compared to their conformations in the MS47134-bound state (Fig. 4A), which results in a more compact pocket than that of MS47134. Given the molecular size of fospropofol is smaller than MS47134, it is likely that the compacted pocket observed in the fospropofol-bound S83-MRGPRX4 is stabilized by fospropofol binding. Along with the inward helical movement, several residues also display distinct rotamer states compared to their conformations in L83-MRGPRX4. Specifically, R82 rotates toward TM3 and occupies a position previously occupied by the phenyl group of MS47134 (Fig. 4B). In addition, S83 moves inwards compared to the position of L83 in L83-MRGPRX4 (Fig. 4C). The observed conformational differences may be partially attributed to the use of distinct ligands. However, we cannot rule out that S83-MRGPRX4 does not share an identical protein conformation as L83-MRGPRX4. In the previously published L83-MRGPRX4 structure, L83 points to the cell membrane. By contrast, S83 in the current study rotates inwards and does not interact with the cell membrane. Given S83 is more hydrophilic than L83, it is likely less stabilized by the lipid milieu as the L83 counterpart. This difference may affect the overall MRGPRX4 conformation.

To test this hypothesis, we examined three of our screened drugs, including fospropofol, fosphenytoin, and dexamethasone phosphate, at both the S83 and L83 alleles. To our surprise, three drugs displayed different pharmacological profiles toward S83 and L83 with an improved potency at S83-MRGPRX4 (Fig. 4D, table S3 and fig. S8). Among other established MRGPRX4 agonists, both S83 and L83 demonstrated equal potency toward DCA (a bile acid) whereas the L83 allele, as previously reported, exhibited greater potency and efficacy toward nateglinide and MS47134 (23) (Fig. 4E, fig. S9 and table S3). These results suggest that the residue difference at position 83 affects the conformational state of MRGPRX4, which is critical for agonist recognition. Moreover, this variation may impact the receptor’s preference for specific ligands with distinct structural or chemical characteristics.

## DISCUSSION

Phosphate modification of drugs frequently improves their solubility, allowing for alternative routes of administration. In addition to dexamethasone phosphate, fosphenytoin is 4500-fold more soluble than phenytoin (40) and fospropofol is 3000-fold more soluble than propofol (41). Unfortunately, these improvements in solubility come with the tradeoff of treatment-limiting pruritus. Of note, in the case of fospropofol, the highest potency ligand identified in our study, bothersome paresthesia/pruritus was cited as a principal factor for its discontinuation (42). The role of MRGPX4 suggests its involvement specifically in pruritus whereas the precise etiology of paresthesia in this context is still unknown. Employing reverse pharmacology with a specific MRGPRX4 antagonist to determine if paresthesia resolves may help elucidate this aspect. In addition to the distressing events, historically, this pruritus has been thought to represent a cutaneous manifestation of allergy which may herald more severe consequences, such as anaphylaxis. However, our findings illuminate a completely different mechanism for phosphor-drug-induced itch: instead of triggering an allergic reaction, these drugs cause itch by directly activating MRGPRX4 in itch-sensing neurons. The implication of this finding is clinically relevant. Pharmacological inhibition of MRGPRX4 can potentially eliminate the irritating sensory side effects without interfering with the medication’s intended purposes. This approach can drastically expand the pharmacological repertoire when dosage escalation by phosphate modification of drugs is considered to reach clinical efficacy. Importantly, our identified mechanism is distinct from allergic itch where histamine is the primary itch mediator that can be countered by antihistamines, commonly employed as first line anti-itch medications.

The conformational plasticity of the MRGPRX4 agonist binding pocket is likely essential for the receptor’s ability to recognize a range of diverse agonists encompassing phosphate-containing drugs of variable shape and size, as well as bile acids and bilirubin (15–17, 23, 30). Our cryo-EM structure of fospropofol-bound MRGPRX4 Gq complex in conjunction with previously published structures help to mechanistically explain this chemical promiscuity. We confirm key amino acid residues, R82, R86, and R95, responsible for phosphate recognition, and clarify structural differences among MRGPRX4 variants. Additionally, our structure provides preliminary insight into the hydrophobic binding pocket of MRGPRX4, which may have clinical relevance. Among tested steroid phosphates, dexamethasone phosphate displayed the highest potency for MRGPRX4, far higher than prednisolone- or hydrocortisone phosphate (table S1.). Based on our structure, we infer that this may be due to the increased hydrophobicity of the steroid nucleus of dexamethasone phosphate, with fluorine substituting hydrogen at C9 and an additional methyl group at C16.

Limitations of this study include the lack of a mouse ortholog. This may partially account for why the itch side effects from phosphomonoester drug usage has long been documented, with the earliest in 1970s (3, 4); however, we have not gained any observation or mechanistic explanation from animal models. To resolve this obstacle, we employed a humanized mouse model which demonstrates scratching behavior mimicking human itching response. Nonetheless, the observed behavioral phenotype may be exaggerated or skewed by the non-physiologic amounts of receptor expression. A recent study identified the presence of human receptor activity-modifying proteins (RAMPs) that modulate MRGPRX4 expression (43). It is unclear if MRGPRX4 can also be regulated by the murine RAMP counterpart. In addition, despite our efforts to rule out itch receptors sharing conserved sequence homology or functionality across humans and mice, we recognize the potential presence of unidentified primate-specific itch receptors. Conceivably, these receptors may possess comparable pharmacology, akin to the MRGPRX family, contributing to phospho-drug-induced itch.

Being the predominant variant of MRGPRX4, the S83 variant represents 99% of variants within the global population. Nevertheless, clinically reported pruritus associated with phosphomonoester prodrugs does not reflect the populational prevalence of the S83 allele. Given our demonstrated structural differences between S83 and the previously published L83, even individual amino acid changes in this highly variable sequence can have profound impacts of receptor activation. Furthermore, RAMP2 was reported to downregulate the surface expression of MRGPRX4 (43). This finding highlights a regulatory mechanism that could also shape the representation of itch. To address these limitations and further translational value, research examining MRGPRX4 variants, their function and structure, potential interactors as well as correlation with clinical pruritus is required.

Our study primarily aimed to identify pharmaceuticals unintentionally targeting MRGPRX4. It is important to note that the current drug library is limited. Additional screens would capture more interactions and uncover the full chemical range of the MRGPRX4 ligand repertoire. Moreover, endogenous phosphorylation signifies pleiotropic biological processes extending beyond energy transfer, signaling transduction, and cell regulation (44, 45). Given the demonstrated pharmacology of MRGPRX4 toward phosphate-modified medications, it will be important to explore its potential interaction with endogenous phosphorylated small molecules. This could illuminate the role of MRGPRX4-mediated pruritus in other pathophysiological processes, perhaps providing explanations for this primate-specific phospho-sensing phenomenon.

Overall, our study demonstrates that MRGPRX4 recognizes a variety of phosphate-modified medications, potentially explaining the mechanism by which these compounds cause bothersome pruritus. We provide a structural resource which can guide rational drug design and *in silico* screens to limit adverse cutaneous drug associations. Our developed preclinical platform presents a potential tool for future antagonist development and optimization. Finally, our data lends additional support for MRGPRX4 as a therapeutic target for non-histaminergic pruritus, especially when the treatment options are limited in the pharmacologic context.

## Material and Methods

### Study Design

The primary objective of this study was to understand the mechanisms underlying pruritus associated with phosphomonoester drugs. Aligned with this objective, each approach and methodology were developed and employed to investigate the phenomena across varying resolutions. Our approach spanned from in vivo mouse behavioral studies demonstrating the causal relationship, to in vitro analyses of cellular calcium dynamics (including mouse primary cultured DRG neurons and HEK293 cell lines), and further down to the structural elucidation of amino acid residues essential for phosphate group recognition. Both behavioral and in vitro experiments were conducted in a blocked manner, accounting for various factors, including treatment, genotype, and different genetic variants of MRGPRX4 (for in vitro studies). Sample sizes for in vitro calcium studies were based on the general standard adopted by the laboratories in the field (numbers of biological replicates are indicated in the figure legends). For behavioral studies, animals were randomly assigned to control and experimental groups. Sample sizes were predetermined by the power analysis and are of sizes similar to previous itch studies (15, 16). Experimenters and subjects scoring the recorded videos were both blind to the mouse genotype and tested compounds.

### Reagents

Propofol was purchased from Adooq Bioscience (Irvine, CA, USA). Fospropofol (Aquavan) was obtained from Toronto Research Chemicals Inc. (Toronto, Ontario, Canada) and The Biotek (Pasadena, CA, USA). Phenytoin sodium and deoxycholic acid were provided by Sigma-Aldrich (St Louis, MO, USA). MS47134 was purchased from MedChemExpress (Monmouth Junction, NJ, USA). U73122 was obtained from Santa Cruz Biotechnology (Dallas, TX, USA), YM-254890 was from Wako Chemicals (Richmond, VA, USA), and thapsigargin was from Tocris (Minneapolis, MN, USA). All other tested compounds were purchased from Cayman Chemical (Ann Arbor, MI, USA).

### Culture of HEK293 cells and generation of stable cell lines

HEK293 human embryonic kidney cells (ATCC) were maintained in growth medium (Dulbecco's Modified Eagle Medium (DMEM) supplemented with 10% heat-inactivated fetal bovine serum (FBS), 100 U/ml penicillin and 100 mg/ml streptomycin) in 5% CO_2_ at 37°C. Stable cell lines expressing human MRGPRX1-X4 were generated as previously described (11, 13, 16, 46). Briefly, HEK293 cells were transfected with the cDNA encoding the human MRGPRs proteins in plasmid using Lipofectamine 3000 (ThermoFisher Scientific). The transfected cells were selected with neomycin or blasticidin in DMEM supplemented with 10% FBS. Each cloned cell was further selected as stable cell lines and confirmed the expression of MRGPRs by evaluating the expression of MRGPR and the activation by their known ligands.

HTLA cells, a cell line derived from HEK293 and engineered to stably express a tTA-dependent luciferase reporter and a β-arrestin2-TEV fusion gene, were generously provided by Richard Axel's laboratory. HTLA cells were cultured in growth medium containing 2 μg/ml puromycin and 100 μg/ml hygromycin B in 5% CO_2_ at 37 °C. Stably MRGPRX4-expressing HTLA cells were generated by transfecting pcDNA3.1(+)-Zeo containing MRGPRX4-Tango construct (either with WT or S83L mutant sequence) into HTLA cells with Lipofectamine 3000 and selecting the successful transfected clones with Zeocin.

### Drug library screens for MRGPRX4 ligands

A collection containing > 3,800 compounds for potential repurposing/repositioning at the ChemCore High Throughput Facility at the Johns Hopkins School of Medicine was used to screen MRGPRX4 agonists. In the initial screen, HEK293 cells vs MRGPRX4-stably expressing HEK293 cells were plated on 100 μg/ml poly-D-lysine (PDL) coated 384-well plate with a seeding density of 1×10^4^ cells/per well. The next day, Calcium 5 dye from the FLIPR Calcium 5 assay kit (Molecular Devices) diluted in imaging buffer (Hank's Balanced Salt Solution (HBSS) with 20 mM HEPES (pH 7.4)) was added to the cells, incubated for 1 h at 37°C, and recovered for 10 min in the dark at room temperature. Then, the stained cells containing plates were tested for intracellular calcium mobilization assay using the Functional Drug Screening System 6000 (FDSS 6000) device (Hamamatsu Photonics). Each well in the 384-well plate was simultaneously imaged using 480 nm excitation/540 nm emission. Data was collected every 1 s for 200 s according to manufacturer's specifications. 20 μM of tested drugs from the compound library were added 20 s after imaging started. We added the imaging buffer as a negative control and MRGPRX4 agonist ursodeoxycholic acid (50 μM) in the same buffer served as a positive control on each testing plate. The relative fluorescence signal at a given time point over the baseline was calculated. The peak response was identified and subtracted with the baseline signal to obtain Max-Min value for each well. To minimize the inter-plate variations, the acquired raw data underwent normalization and adjustment using the averages of negative control and positive control wells on each individual plate. Next, z-score transformations were applied to center each plate across the whole dataset. The tested compounds found to activate control HEK293 cells (lacking MRGPRX4) were further excluded from the analysis.

### EC_50_ determination

HEK293 cells and MRGPRX4 stably expressed HEK293 cells were seeded in 100 μl culture media on 96-well plates at a density of 40,000 cells per well and incubated overnight. The next day, the media were replaced with a dye-loading solution from the FLIPR Calcium 5 assay kit (Molecular Devices), diluted in Hank's Balanced Salt Solution (HBSS) with 20 mM HEPES, pH 7.4. After 1 h of incubation at 37°C, cells were allowed to recover for 10 min in the dark at room temperature prior to performing intracellular calcium mobilization assay in a Flexstation-3 instrument with SoftMax^®^Pro 7.1 software (Molecular Devices). Serial concentrations of tested drugs were prepared in HBSS/HEPES solution at 3X concentration. Responses were monitored as fluorescence intensity at 480 nm excitation/540 nm emission every 2 s with simultaneous data collection for additional 180 s, and 50 μl of drugs were added at 20 s after imaging started. Compounds were tested in triplicates and the signals were averaged. Responses were determined by subtracting the minimum signal (at the baseline before adding the drug) from the maximum signal (obtained after drug stimulation). Four-parameter logistic (4PL) curve fit was used to plot the dose-response curves. The values of EC_50_s (half maximal effective concentration) were determined as the concentration of the substances that gives 50% response of normalized peak response. Both dose-response curve fitting and EC50s calculation are computed with GraphPad Prism software (version: 6.o or above).

### Transient transfection of Murine Mrgprs or MRGPRX4 (including alanine mutants)

To generate murine Mrgprs expressing cells, HEK293T cells (ATCC) were seeded on poly-D-lysine coated 96 well plate at 25,000 cells per well one day prior to transfection. HEK293T cells were transiently co-transfected with the mammalian expression plasmids carrying cDNA encoding the murine Mrgpr genes and plasmid inserted with cDNA encoding murine α-subunit of the heterotrimeric G15 protein (Gα15) in triplicates using Lipofectamine LTX reagents (Thermo Scientific). To generate WT, S83L, and alanine mutant MRGPRX4-expressing cells, HEK293T cells were seeded on poly-D-lysine coated 384 well plate at 12,000 cells per well one day prior to transfection. Mammalian expression plasmids pLX304 (vector from Addgene) carrying different MRGPRX4 coding sequences were transiently transfected into HEK293T cells in triplicates with Lipofectamine LTX reagents (Thermo Scientific). Intracellular calcium mobilization assays were performed after one day of transfection. Murine Mrgprs tested include Mrgpra1, Mrgpra2a, Mrgpra2b, Mrgpra3, Mrgpra4, Mrgpra6, Mrgpra9, Mrgpra10, Mrgpra11, Mrgpra12, Mrgpra14, Mrgprb5, Mrgprb6, Mrgprb10, Mrgprb11, and Mrgprc11. WT and mutant MRGPRX4 constructs tested include WT, S83L, R82A, R86A, R95A, W158A, Y250A, and Y254A.

### Calcium imaging in HEK293 cells

Various HEK293 cell lines were plated on PDL-coated glass coverslips. After overnight culture, cells were loaded with Fura 2-acetomethoxy ester calcium indicator (0.5 μM, Molecular Probes) along with Pluronic F-127 dispersing agent (0.1%, Molecular Probes) for 30 min in the dark at 37°C, then washed with calcium imaging buffer (CIB; NaCl 125 mM, KCl 3 mM, CaCl_2_ 2.5 mM, MgCl_2_ 0.6 mM, HEPES 10 mM, glucose 20 mM, NaHCO_3_ 1.2 mM, sucrose 20 mM, brought to pH 7.4 with NaOH). Drugs were added to Fura 2-loaded cells and intracellular free calcium was measured using 340 nm and 380 nm excitation wavelengths with emission measured at 520 nm with a microscope-based imaging system (Nikon Eclipse TE200). Changes in emission fluorescence ratios at 340/380 nm excitation were continuously monitored at 1 s interval. Relative change in fluorescence intensity at a given time point (F_T_) compared to average baseline prior to adding the tested compounds (F_0_) was calculated and expressed as F_T_/F_0_ representing alteration in intracellular calcium concentration. For pharmacological blocker experiments, cells were pre-incubated with signaling pathway inhibitors, including YM254890, U73122, and thapsigargin, for 30 min before imaging.

### PRESTO-Tango assay

HTLA cells stably expressing either WT or S83L MRGPRX4 were seeded in PDL-coated 96-well white clear bottom cell culture plates with DMEM containing 10% FBS, at a density of 35,000 cells per well and were allowed to attach to the bottom of the wells overnight. The next day, growth medium was replaced with 100 μl of serum-free medium containing the tested drugs at various concentrations, and the cells were then incubated at 37°C for 24 h. On the following day, media with drug solutions was removed and 50 μl per well of Bright-Glo^™^ Reagent from Bright-Glo^™^ Luciferase Assay System (Promega) was loaded. The plate was incubated at room temperature in the dark for 5 min, and the relative luminescence units were measured using the Flexstation-3 instrument with SoftMax^®^Pro 7.1 software (Molecular Devices). Dose response curves and EC_50_s were analyzed as described above.

### Site-directed mutagenesis

To generate MRGPRX4 mutant constructs, site-directed mutagenesis was performed using Q5^®^ Site-Directed Mutagenesis Kit (New England Biolabs). The primers were designed by the NEBaseChanger tool, with forward primers spanning designated mutated sequence corresponding to the desired amino acid substitutions. The plasmids with WT MRGPRX4 sequence served as the templates for polymerase chain reaction (PCR) amplification with Q5 high-fidelity polymerase and respective primer pairs according to the manufacturer’s conditions. Then, the linear PCR products underwent phosphorylation, ligation, and simultaneous degradation of template DNA by incubating them with a KLD enzyme mix consisting of T4 polynucleotide kinase, T4 DNA ligase, and DpnI at room temperature for 5 min. Subsequently, the mutated circular plasmids were transformed into NEB 5-alpha competent *E. coli* by heat-shock method, and the successfully transformed bacteria were selected on LB plates with selection antibiotics. The next day, the transformant colonies were picked, expanded in LB broth containing selection antibiotics overnight. Plasmids were isolated using QIAprep Spin Miniprep Kit (Qiagen) and subjected to sequencing to confirm the mutation.

### Animal care and use

All experiments were carried out following the protocols approved by the Animal Care and Use Committee at The Johns Hopkins University School of Medicine. The Johns Hopkins University IACUC meets the requirements of the Federal Law and NIH animal use regulations. Animals (5 mice per cage at maximum) are housed in ventilated racks with proper temperature, humidity, and 12 h light/dark cycle control.

### Generation of animal models

All mice used were in C57BL/6J background or have been backcrossed to C57BL/6J mice for at least 5 generations. All the animals used were heterozygous unless otherwise specified. NP2-X4+ and NP2-WT littermates were generated by crossing *Mrgpra3-Cre* to *Rosa26-lsl-MRGPRX4* as detailed previously (15). NP2-X4+ mice were further crossed to homozygous *Rosa26-lsl-GCaMP6s* to obtain NP2-WT/GCaMP6s and NP2-X4+/GCaMP6s animals. A detailed mating strategy is illustrated in fig. S5. *Pirt-Cre-GCaMP6s* animals were acquired by crossing *Pirt-Cre* to homozygous *Rosa26-lsl-GCaMP6s*. *Pirt-Cre* animals were generated through homologous recombination as indicated previously (37). *Rosa26-lsl-GCaMP6s* mouse line was provided by Dr. Dwight Bergles’ lab at the Johns Hopkins School of Medicine.

### Itch behavioral studies

Behavioral assays were conducted in accordance with previously established protocols (15, 16). Experimenters and subjects scoring the recorded videos were both blind to the mouse genotype and tested compounds. Behavioral tests comparing NP2-WT and NP2-X4+ scratching behavior were 6–12weeks old littermates including both genders. In brief, on the day preceding the experiment, animals were acclimated to the test chamber for at least 30 min and the experimenters performed mock injections 1–2 times in between. On the day of experiments, animals were habituated in test chambers for 10 min before receiving subcutaneous injection of 50ul compounds. Videos were recorded for 30 min after the injection. A bout of scratching was defined as a sustained scratching movement initiated by hindpaws toward the injection site (until the pause of the movement / withdrawal of the hindpaw). Scratching bouts were quantified within the 30 min record sessions.

### DRG dissociation and culture

The procedures were performed following previously established protocols (15). Murine DRGs (from both male and female) were harvested from all spinal segments and digested in dispase (5 mg/ml) / type I collagenase (1 mg/ml) enzyme mix at 37 °C for an hour. Enzyme mix was replaced with complete DH10 media (90% DMEM/F12, 10% FBS, penicillin (100 units/ml), and streptomycin (100 μg/ml)) and gently triturated with 1 ml pipette tip until the media turned cloudy without visible tissue chunks. Dissociated tissues were filtered through a 70 μm filter and spun down 1250 rpm for 5 min at RT. Supernatant was removed and the cell pellet was resuspended in complete DH10 media. Cell suspension was then added on top of poly-D-lysine- and laminin-coated coverslips and sat for 1–2 h at 37°C. Complete DH10 media supplemented with NGF (50 ng/ml) was then added into the vessel. Neurons were allowed to recover overnight at 37°C for the next-day calcium imaging experiment.

### Neuron calcium imaging and analysis

Calcium imaging data was collected with a Nikon Eclipse TE 200 microscope. Time series GCaMP6 fluorescence intensity was acquired through the FITC/GFP channel. Before the imaging, cultured neurons were washed and equilibrated in CIB for 30 min at 37°C. In each trial, compounds (as indicated in the results) were added into the imaging chamber after a 30 s baseline GCaMP6 activity was collected. For serial additions of compounds, washouts with CIB were performed in between. Chloroquine or KCl was used as the positive controls to ensure the viability and subtypes of the neurons. Neurons with GCaMP6 signal rose above 50% of the baseline intensity after compound application were counted as positive responders and the percentage of responding neurons was calculated. Neurons meeting the following descriptions were excluded: high or unstable baseline GCaMP6 activity, loosely attached to the coverslips, or GCaMP6 activity unable to return to baseline after washouts.

### In situ hybridization

In situ hybridization of mouse DRGs with a custom *MRGPRX4* probe was performed on 16–20 μm cryo-sections using the HCR RNA-FISH platform (Molecular Instruments), following the protocol and instructions from the manufacturer. Nuclei were counterstained with DAPI. Images were acquired with Zeiss LSM-800 confocal microscope at 20x. The custom probe for *MRGPRX4* was designed by the manufacturer to avoid cross-reactivity to the mouse genome.

### The construct, expression, and purification of S83-MRGPRX4-Gq complex

The S83 MRGPRX4 pFastBac1 construct for protein expression was generated by site-directed mutagenesis using the previously reported MRGPRX4 construct (23). For Gq and scFv16, we used the same mini-GαqiN heterotrimer and scFv16 reported in the previous MRGPRX4 publication (23). The viruses were generated using a Bac-to-Bac Baculovirus Expression System (Invitrogen). For the MRGPRX4-Gq complex, MRGPRX4 and mini-GαqiN heterotrimer were co-expressed by infecting *sf9* cells at a density of 2 million cells per ml with viruses at multiplicity of infection (MOI) ratio of 3:1. Cells were harvested 48 h after infection and stored in −80 °C until further use. The day of purification, the cells expressing MRGPRX4-Gq complex were thawed on ice and incubated with a buffer containing 20 mM HEPES, 50 mM NaCl, 1mM MgCl2 and proteinase inhibitor at room temperature. After 1 h, the cells were homogenized. The cell membranes were collected by centrifugation at 25,000 rpm for 30 min using a Ti45 rotor (Beckman) and solubilized using a buffer containing 20 mM HEPES, 100 mM NaCl, 5% (w/v) glycerol, 0.6% (w/v) lauryl maltose neopentyl glycol (LMNG), 0.06% (w/v) cholesteryl hemisuccinate (CHS) for 5 h at 4 °C. After centrifugation at 32,000 rpm for 30 min using a Ti70 rotor (Beckman), the supernatant was applied to a 50 ml conical tube and incubated overnight at 4 °C with 1 ml Talon IMAC resin (Clontech) and 20 mM imidazole. The resin was collected the next day and washed with 25 column volumes buffer containing 20 mM HEPES, 100 mM NaCl, 5% (w/v) glycerol, 0.01% (w/v) LMNG, 0.001% (w/v) CHS and 30 mM imidazole. The MRGPRX4-Gq complex was then eluted with a buffer containing 20 mM HEPES, 100 mM NaCl, 5% (w/v) glycerol, 0.01% (w/v) LMNG, 0.001% (w/v) CHS and 250 mM imidazole. The eluted protein sample is concentrated and subjected to size-exclusion chromatography on a Superdex 200 Increase 10/300 column (GE Healthcare) that was pre-equilibrated with a buffer containing 20 mM HEPES pH7.5, 100 mM NaCl, 0.00075% (w/v) LMNG, 0.00025 (w/v) glyco-diosgenin (GDN), 0.00075% (w/v) CHS and 100 μM TCEP. The peak fractions were concentrated to 3–5 mg/ml and incubated with 200 μM fospropofol for 2 h prior to grid-making.

### CryoEM data collection and 3D reconstitution

3.2 μl fospropofol-bound MRGPRX4-Gq sample was applied to glow discharged Quantifoil R1.2/1.3 Au300 holey carbon grids (Ted Pella) and flash frozen in a liquid ethane/propane (40/60) mixture using a Vitrobot mark IV (FEI) set at 4 °C and 100% humidity with a blot time range from 3 to 4.5 s. Images were collected using a 200 keV Talos Artica with a Gatan K3 direct electron detector at a physical pixel size of 0.88 Å. Movies were semi-automatically recorded using SerialEM as previously described (47, 48). Particles from a manually curated dataset were selected using Blob particle picker within the cryoSPARC package (49, 50). A subset of these particles was used to train Topaz and the particles were repicked from the micrographs (51). The combined particles were then subjected to 2D or 3D classification to resolve a final stack of particles that produced a map with resolutions reported in table S2 (by FSC using the 0.143 Å cut-off criterion) (52). Additional sharpening was carried out using deepEMhancer (53). For more details see fig. S7.

### Model building and refinement

The fospropofol-bound MRGPRX4-Gq map generated from deepEMhancer (53) was used for model building, refinement, and subsequent structural interpretation. The models of MRGPRX4, miniGq heterotrimer and scFv16 taken from the MRGPRX4-Gq complex (PDB: 7S8P) were docked into the fospropofol-bound MRGPRX4-Gq map using ChimeraX (54). As the S83 variant of MRGPRX4 was used in this study, we mutated L83 to S83 before the refinement. The initial model was manually refined in Coot (55) and then subjected to several rounds of real-space refinement in Phenix (56) and manually refinement in Coot to obtain the final model. The model statistics were validated using Molprobity (57). The structural figures were prepared by either ChimeraX or PyMOL (https://pymol.org/2/). The detailed refinement statistics are provided in table S2.

### Statistical analysis

Statistical tests were performed and visualized using Prism software (Graphpad). All data were presented as means ± SEM unless otherwise indicated. All animals or samples that underwent successful procedures or treatments were included in the analyses. Statistical significance for the behavioral tests was established using two-tailed unpaired Student’s t tests with a threshold of p < 0.05. For behavioral studies, sample size was predetermined by power analysis. To compare EC_50_ fits for in vitro calcium mobilization assay results, the extra sum-of-square F test was employed to examine the difference in EC_50_s. The null hypothesis assumed that the EC_50_ was the same for the datasets, with p-value cutoff at 0.05. In the case of nateglinide, due to unsuccessful curve fitting for the WT, one-sided t-test was used as an alternative statistical test.

## Supplementary Material

supplemental data

table

MDAR

## Data Availability

All data are available in the main text or the supplementary materials. The coordinate and cryo-EM maps of fospropofol-bound MRGPRX4-Gq complex have been deposited to PDB (EMDB) database with accession codes 8YRG and EMD-39542, respectively. The raw cryo-EM micrographs of fospropofol-bound MRGPRX4-Gq complex have been deposited to the EMPIAR database (https://www.ebi.ac.uk/empiar/) with accession numbers EMPIAR-11986. Plasmids, cell lines, and mouse lines used in this study will be provided upon request to the co-corresponding author (X.D.) under a material transfer agreement with Johns Hopkins University School of Medicine.
