# Supplementary material for: MRGPRX4 mediates phospho-drug associated pruritus in a humanized mouse model
Source: Sci Transl Med. Author manuscript; Available in PMC 2024 Dec 14. (PMC11645656; doi:10.1126/scitranslmed.adk8198)
Supplement: supplemental data [file NIHMS2038790-supplement-supplemental_data.pdf]

Science Translational Medicine
AAAS

Supplementary Materials for

MRGPRX4 mediates phospho-drug–associated pruritus in a humanized mouse model

Daphne Chun-Che Chien *et al.*

Corresponding author: Jonathan F. Fay, jfay@som.umaryland.edu; Bryan L. Roth, bryan_roth@med.unc.edu; Xinzhong Dong, xdong2@jhmi.edu

Sci. Transl. Med. **16**, eadk8198 (2024)
DOI: 10.1126/scitranslmed.adk8198

The PDF file includes:

Figs. S1 to S9
Tables S1 to S3

Other Supplementary Material for this manuscript includes the following:

Data file S1
MDAR Reproducibility Checklist

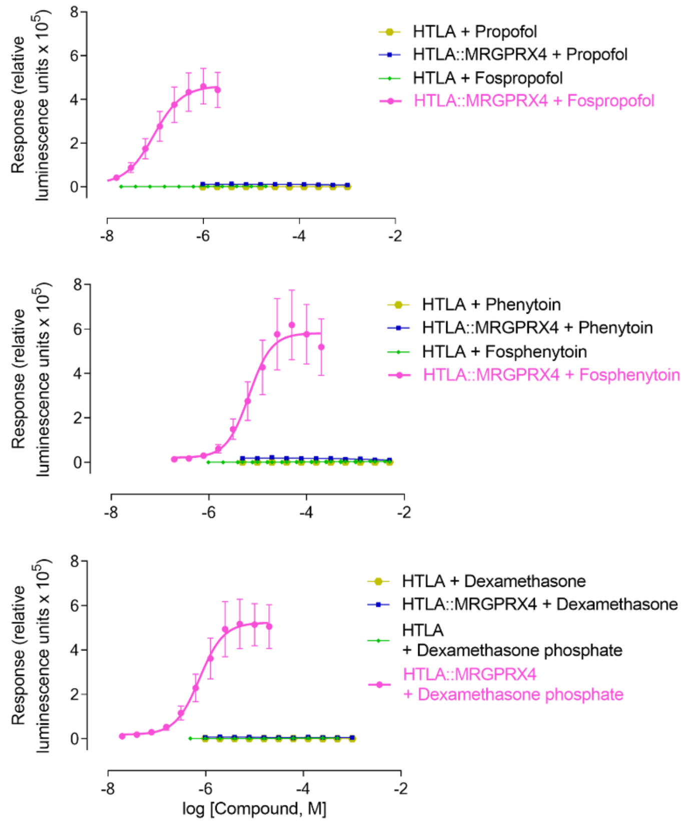

fig. S1. Phosphate monoester prodrugs activate human MRGPRX4 by β-arrestin-dependent PRESTO-Tango assay. Dose-response curves of fospropofol (*top*), fosphenytoin (*middle*), dexamethasone phosphate (*bottom*) with their parental drug against MRGPRX4 by β-arrestin (Tango) recruitment assay. Data are from at least two independent experiments, each performed in triplicate, and are depicted as mean ± SEM. HTLA: HEK 293 cell line stably expressing both a β-arrestin2-TEV fusion gene and a tTA-dependent luciferase reporter.

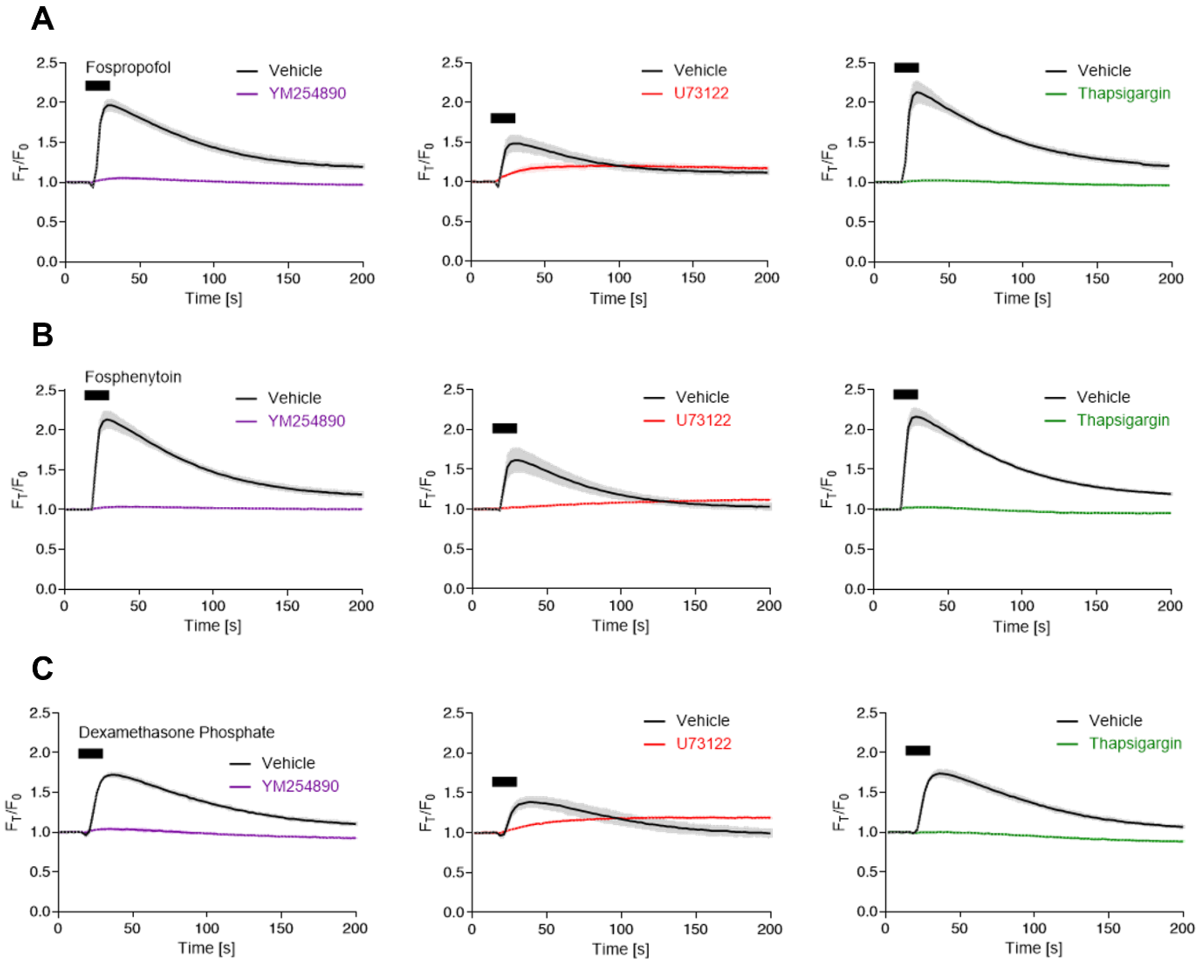

fig. S2. Phosphate monoester prodrugs activate MRGPRX4 through Gαq signaling pathway. Traces of $[Ca^{2+}]_i$ from MRGPRX4-expressing HEK293 cells stimulated with (**A**) 1 µM fospropofol, (**B**) 10 µM fosphenytoin, (**C**) 1 µM dexamethasone phosphate at 20 s. Cells were preincubated with either 10 µM of the Gαq inhibitor YM 254890 (*left*), 20 µM PLC inhibitor U73122 (*middle*), or 10 µM sarco/endoplasmic reticulum Ca2+-ATPase inhibitor thapsigargin (*right*) for 30 min before imaging. Data are shown as mean ± 95% confidence interval of two independent experiments, each performed in quadruplicate.

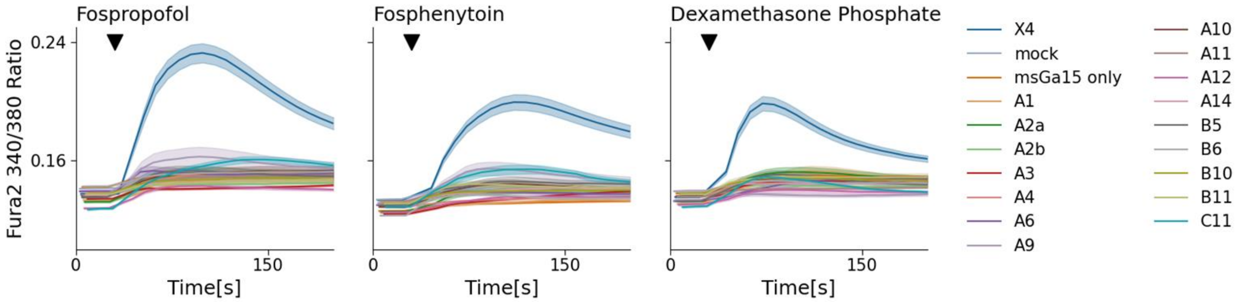

fig. S3. Phosphate monoester prodrugs do not activate mouse Mrgprs. Averaged fura-2 fluorescence 340/380 ratio traces of individual HEK293 cells transiently expressing MRGPRX4 or individual mouse Mrgpr together with mouse (ms) heterotrimeric Gα15 exposed to 10 μM fospropofol (*left*), fosphenytoin (*middle*) or dexamethasone phosphate (*right*). After 30 s baseline was established, compound was added at the time indicated by arrowheads. Data are reported as mean ± 95% confidence interval (shaded areas) with n = 150-230 cells in each Mrgpr vs. treatment group, pooled from three independent experiments. X4: human MRGPRX4. Individual mouse Mrgpr construct is abbreviated as in parentheses, including Mrgpra1 (A1), Mrgpa2a (A2a), Mrgpra2b (A2b), Mrgpra3 (A3), Mrgpra4 (A4), Mrgpra6 (A6), Mrgpra9 (A9), Mrgpra10 (A10), Mrgpra11 (A11), Mrgpra12 (A12), Mrgpra14 (A14), Mrgprb5 (B5), Mrgprb6 (B6), Mrgprb10 (B10), Mrgprb11 (B11) and Mrgprc11 (C11).

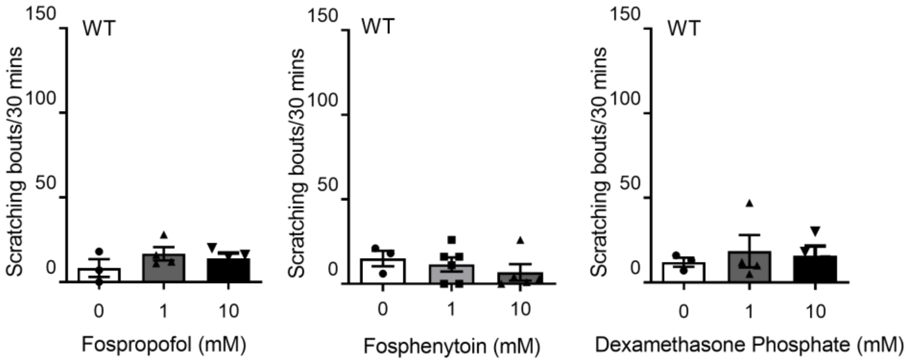

fig. S4. Phosphate monoester prodrugs are not pruritogenic in WT mice. Scratching bouts recorded from WT mice underwent subcutaneous nape injection of fospropofol (*left*), fosphenytoin (*middle*), or dexamethasone phosphate (*right*) across different concentrations (0, 1, or 10 mM) (n = 3-6 mice in each condition). Mean ± SEM as depicted.

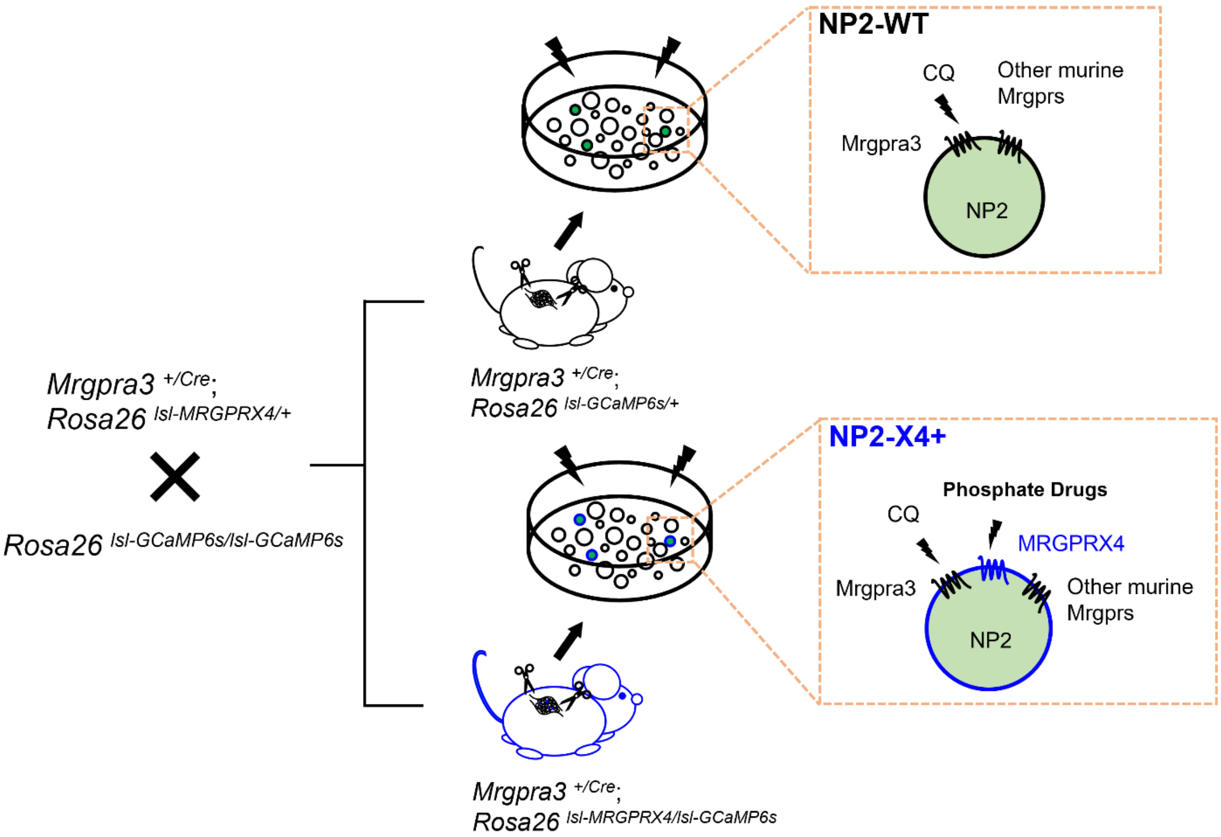

fig. S5. Genetic strategy to label murine NP2 neurons with GCaMP6s. GCaMP6s expression is depicted in green.

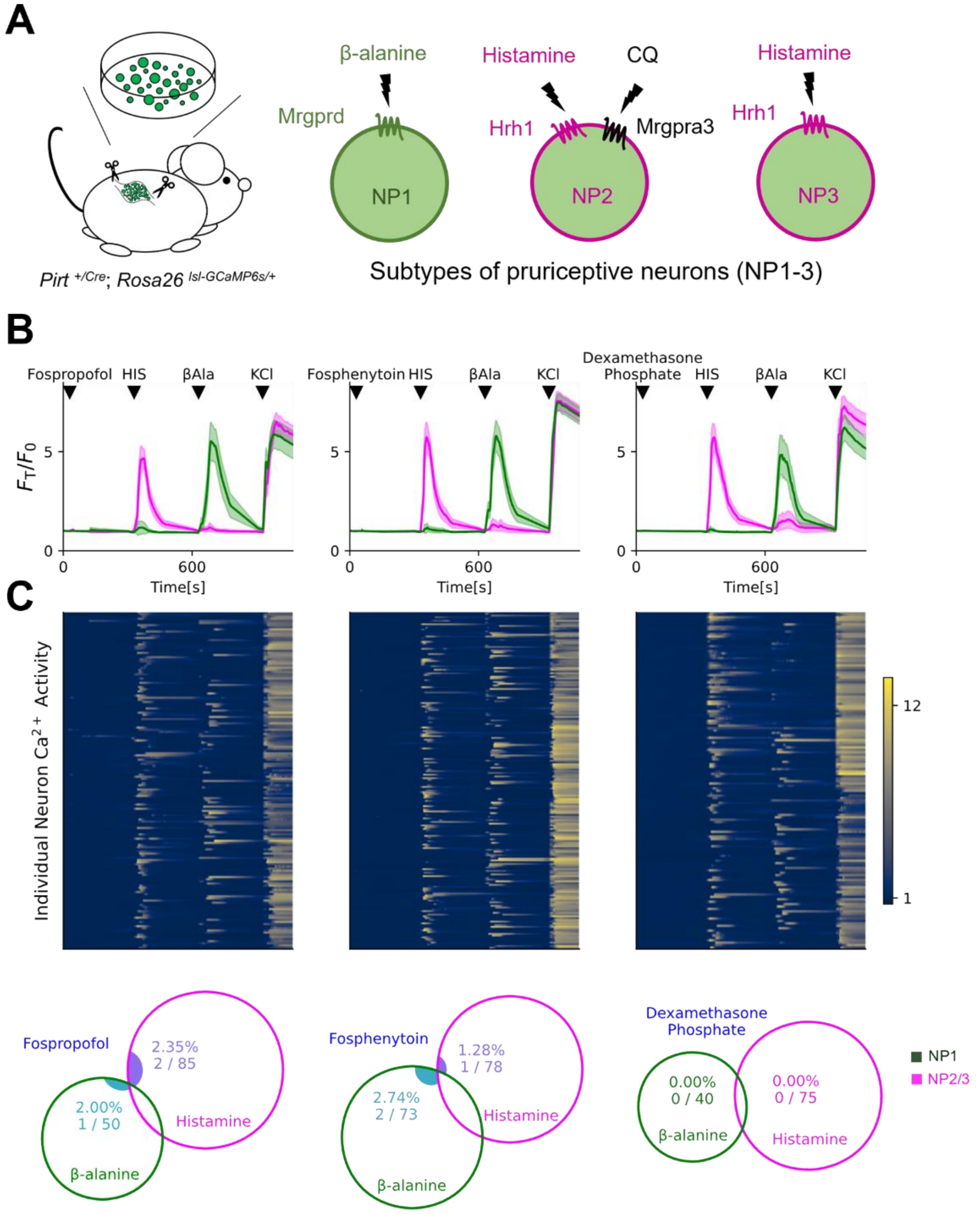

fig. S6. Phosphate monoester prodrugs do not activate WT NP1-3 neurons. **(A)** *Left*: Diagram showing the *in vitro* dissociated DRG culture from Pirt-Cre; Rosa-lsl-GCaMP6s mice. *Right*: various pruritogen-receptor pairs mediating itch through NP1-3 neurons. **(B)** Traces of averaged (*top*) and individual (*bottom*) calcium activity, measured by fluorescence intensity normalized to baseline intensity (F_T/ F_0) over time from NP1-3 DRG neurons labelled with GCaMP6s. Averaged responses of β-alanine-sensitive neuron (NP1, in green) and histamine-sensitive neurons (NP2 and

3, in magenta) were depicted as mean ± 95% confidence interval (shaded areas). Individual $[Ca^{2+}]_i$ activity from the recorded neurons is shown in heatmap. Each row represents the response of one neuron. Compounds were added as indicated by the arrowheads. 10 μM fospropofol, fosphenytoin or dexamethasone phosphate, 50 μM histamine, 1 mM β-alanine and 50 mM KCl were used. $F_T/F_0 > 1.5$ was considered an activation. **(C)** Venn diagram showing the percentage of fospropofol- (*left*), fosphenytoin- (*middle*), or dexamethasone phosphate- (*right*) responsive neurons in NP1 (green) or NP2/3 (magenta) populations. Number of neurons was shown below the percentage, depicted as (number of neurons responded to phospho-drugs) / (number of neurons responded to either βAla or HIS). For each phospho-drug, data are pooled from 3-4 independent culture vessels from 2 mice. NP, non-peptidergic; βAla: β-alanine; HIS: histamine. Hrh1: H1 histamine receptor.

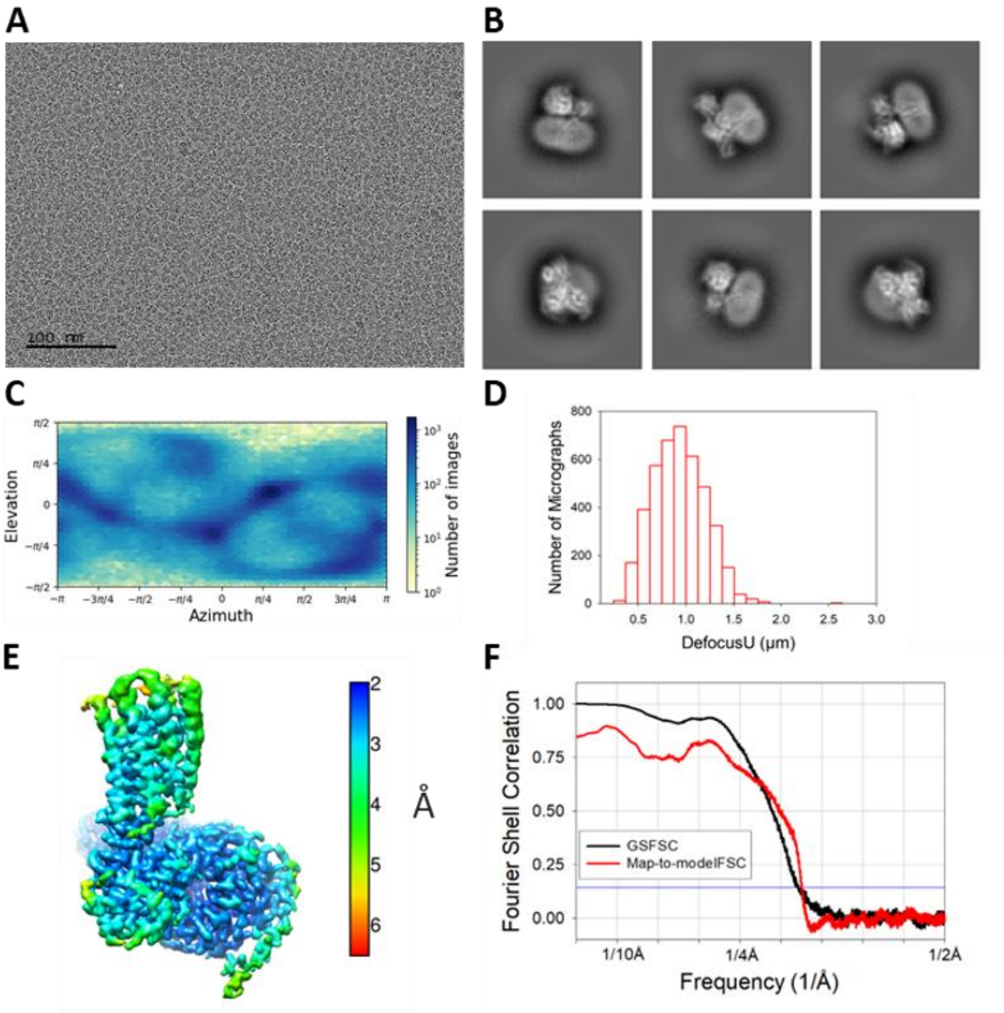

fig. S7. Cryo-EM images and data processing of fospropofol bound S83-MRGPRX4-Gq complex. (**A**) A selected frame aligned micrograph. (**B**) Selected 2D class averages (**C**) Orientational distribution heat map (**D**) Histograms of defocus values for micrographs used in the single-particle analysis. (**E**) Local resolution heat-map calculated using the local windowed FSC method. (**F**) 2D plots of the gold-standard Fourier shell correlation (GSFSC) between half maps (black) and FSC between model and the B-factor sharpened map for respective refined model (red) as calculated by phenix.mtirage.

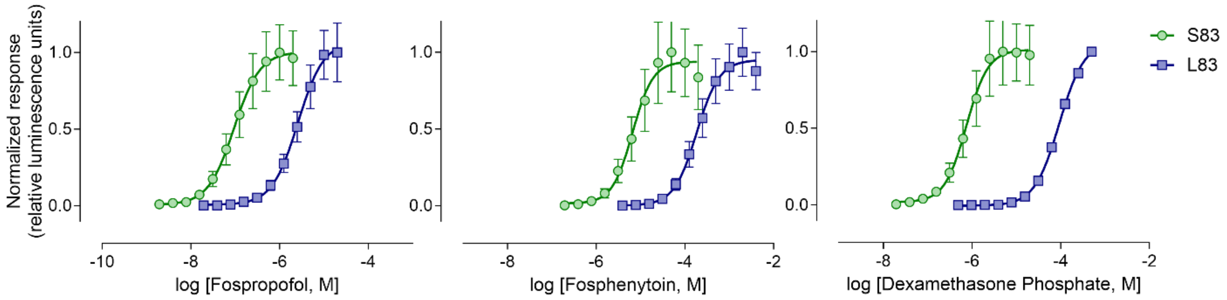

fig. S8. S83-MRGPRX4 is more sensitive to phosphate monoester prodrugs than L83-MRGPRX4 variant as measured by PRESTO-Tango assay. Dose-response curves of fospropofol (*left*), fosphenytoin (*middle*), or dexamethasone phosphate (*right*) against S83 vs. L83-MRGPRX4 by β-arrestin (Tango) recruitment assay. Data are from three independent experiments, performed in triplicate, and depicted as mean ± SEM.

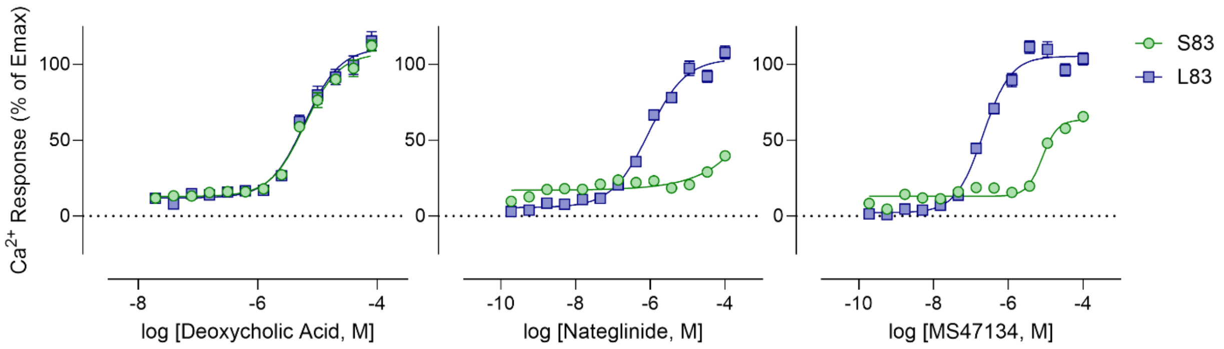

fig. S9. Comparison of S83 and L83-MRGPRX4 variants toward non-phosphorylated MRGPRX4 agonists. Dose-response curves of deoxycholic acid (*left*), nateglinide (*middle*), or MS47134 (*right*) against S83- vs. L83-MRGPRX4 measured by calcium mobilization assay. Data are from three independent experiments, each performed in triplicate and depicted as mean ± SEM.

table S1. Dose-response curve fitting parameters of MRGPRX4 toward phosphomonoester drugs, parental drugs, and non-phosphorylated derivatives.

Compound	EC_{50} (nM)		Hill coefficient	
	value	95% CI	value	95% CI
Fosphenytoin	**77.01**	[52.63 – 115.10]	1.917	[0.61 – 3.23]
Phenytoin sodium*				
(*S*)-mephenytoin				
(*R*)-mephenytoin				
Hydroxyphenytoin				
Phenylethylhydantoin				
Fospropofol	**3.78**	[1.82 – 6.78]	0.800	[0.33 – 1.27]
Propofol*				
Propofol sulfate				
Propofol β-D-glucuronide				
Dexamethasone phosphate	**14.68**	[5.44 – 22.10]	1.652	[-0.096 – 2.21]
Dexamethasone*				
Dexamethasone acetate				
Dexamethasone palmitate	**60970**	[39370 – 99360]	1.502	[0.54 – 2.46]
Dexamethasone 9,11-epoxide				
6β-hydroxy Dexamethasone				
Hydrocortisone sodium phosphate	**327.7**	[139.8 – 925.0]	1.411	[-0.37 – 3.20]
Hydrocortisone 21-hemisuccinate	EC_{50} > 500 μM			
Prednisolone phosphate	**267.1**	[149.6 – 588.2]	0.753	[0.47 – 1.04]
6α-Methylprednisolone 21-hemisuccinate				
Tedizolid phosphate	**157.2**	[73.91 – 461.10]	1.352	[-0.24 – 2.95]
Tedizolid*				
Fosaprepitant dimeglumine	**7003**	[4381 – 8813]	3.670	[-1.53 – 8.87]
Aprepitant*				

*Parental drug
Note: Absence of values in certain cells represents instances where curve fitting could not be performed or did not converge successfully when the highest concentration of compounds at 500 μM was used.
CI: confidence interval.

table S2. Cryo-EM data collection, refinement and validation statistics

Fospropofol-bound MRGPRX4-Gq complex (EMD-39542) (PDB 8YRG)	
**Data collection and processing**	
Magnification	45,000
Voltage (kV)	200
Electron exposure (e–/Å^2)	56.5
Number of movies used	4202
Defocus mean (SD) µm[1]<br>Min-Max	0.9 (0.3)<br>0.2-2.8
Pixel size (Å)	0.88
Symmetry imposed	C1
Initial particle images (no.)	759,190
Final particle images (no.)	375,325
Map resolution (Å)[2]<br>FSC threshold	3.14<br>0.143
Map resolution range (Å)	2.7-5.7
**Refinement**	
Initial model used (PDB code)	7S8P
Model resolution (Å)<br>FSC threshold	3.23<br>0.5
Map sharpening B factor (Å2)	109.6
Model composition	
Non-hydrogen atoms	7977
Protein residues	1079
Ligands	1
B factors (Å^2)	
Protein	63.94
Ligand	84.12
R.m.s. deviations	
Bond lengths (Å)	0.007
Bond angles (°)	0.675
Validation	
MolProbity score	1.94
Clashscore	8.99
Poor rotamers (%)	0.00
Ramachandran plot	
Favored (%)	92.65
Allowed (%)	7.35
Disallowed (%)	0.00

[1] underfocus positive

table S3. Dose-response curve fitting parameters of various agonists toward MRGPRX4 WT (S83), alanine mutants, and S83L variant.

Compound	MRGPRX4	Hill coefficient		EC_{50} (nM)		Statistical test
		value	95% CI	value	95% CI	EC_{50} is different from the WT EC_{50} (p value)
Fospropofol	WT	0.82	[0.62 - 1.06]	29.2	[20.89 - 39.93]	
	R82A	1.92	[1.2 - 2.94]	26369.8	[20059.48 - 43053.55]	Yes (p< 0.0001)
	R86A					
	R95A					
	W158A					
	Y250A					
	Y254A					
	S83L	0.96	[0.72 - 1.29]	156.73	[114.99 - 216.12]	Yes (p< 0.0001)
Fosphenytoin	WT	0.67	[0.53 - 0.83]	1455.83	[1070.55 - 2016.52]	
	R82A					
	R86A					
	R95A					
	W158A					
	Y250A					
	Y254A					
	S83L	0.92	[0.67 - 1.25]	20075.54	[14122.5 - 32147.82]	Yes (p< 0.0001)
Dexamethasone Phosphate	WT	0.77	[0.58 - 1.01]	166.86	[118.42 - 242.68]	
	R82A					
	R86A					
	R95A					
	W158A					
	Y250A					
	Y254A					
	S83L	1.07	[0.76 - 1.48]	5172.07	[3648.77 - 8408.18]	Yes (p< 0.0001)
Deoxycholic acid	WT	1.47	[1.13 - 1.91]	6329.2	[5216.51 - 8041.69]	
	S83L	1.51	[1.1 - 2.07]	5934.22	[4810.21 - 7771.42]	No (p= 0.6839)
Nateglinide	WT					
	S83L	0.87	[0.71 - 1.06]	895.29	[712.81 - 1150.16]	Yes (p< 0.0001)
MS47134	WT	2.09	[1.28 - 3.3]	7995.43	[6530.94 - 10277.51]	
	S83L	1.18	[0.98 - 1.42]	211.42	[173.81 - 257.39]	Yes (p< 0.0001)

Note: Absence of values in certain cells represents instances where curve fitting could not be performed or did not converge successfully.
CI: confidence interval.
